# Chloroform

**Published:** 1854-07

**Authors:** James Harrison

**Affiliations:** Dentist, Jamestown.


### ARTICLE VI.

*Chloroform.* Letter from JAMES HARRISON, Dentist, Jamestown.

ENCLOSED I send you, my method of counteracting unfavorable impressions made by chloroform, or the means I use when a person is sinking under the effect of chloroform. I have read a case in the American Journal of Dental Science, page 272, vol. 4. The brain, the centre of the nervous system, should be immediately aroused to action, and the valves of the heart put in motion again. The remedy is this: sicken or nauseate the stomach, mechanically as follows: take a light whole goose quill, run the feather end down the throat of your patient to the larynx, give the quill a rotary motion, or if there is not any thing more convenient, take your finger, run it down the throat, move it round and round the side of the throat, touching or exciting the larynx, and mostly the epiglottis. The quill would be preferable if the jaws are locked, if so, run the feather end of the quill between the teeth or back of them, reaction will immediately take place.

I would as soon think of restoring a person sinking under the effect of chloroform by tickling the sole of the foot as to depend on artificial respiration alone. I am confident that this remedy will be of great use to suffering humanity. May it be fairly tested, and endorsed by all who find it to be of use.

N. B. I never let the pulse fall below 45 to the minute—consider the patient safe, pulse at 50.

[An operation like this was recommended, by Ricord, who believed death to take place in consequence of a closure of the glottis. He thrust his hand down the throat, and lifted the epiglottis, which he always found pressed down upon the upper opening of the larynx. This treatment was highly approved and very generally adopted after its first introduction.—*Eds.*]

